# Supplementary material for: The transcription factor ATF3 switches cell death from apoptosis to necroptosis in hepatic steatosis in male mice
Source: Nat Commun. 2023 Jan 23;14:167. doi: 10.1038/s41467-023-35804-w (PMC9871012; doi:10.1038/s41467-023-35804-w)

Supplementary Information for

The transcription factor ATF3 switches cell death from apoptosis to necroptosis in hepatic steatosis in male mice

Yuka Inaba, Emi Hashiuchi, Hitoshi Watanabe, Kumi Kimura, Yu Oshima, Kohsuke Tsuchiya, Shin Murai, Chiaki Takahashi, Michihiro Matsumoto, Shigetaka Kitajima, Yasuhiko Yamamoto, Masao Honda, Shun-Ichiro Asahara, Kim Ravnskjaer, Shin-ichi Horike, Shuichi Kaneko, Masato Kasuga, Hiroyasu Nakano, Kenichi Harada, Hiroshi Inoue

Contents

Supplementary Methods
Supplementary Table 1. Antibodies for immunoblotting and immunohistochemistry
Supplementary Table 2. Specific primer sequences used for quantitative PCR
Supplementary Table 3. Sequences of various lengths and their mutants used to construct the Ripk3 promoter
Supplementary Table 4. Antisense probe sequences for in situ hybridisation
Supplementary Table 5. Baseline characteristics by stage
Supplementary Fig. 1. Non-apoptotic cell death is increased in severe steatosis after hepatectomy
Supplementary Fig. 2. Non-apoptotic cell death due to necroptosis in severe steatosis after hepatectomy
Supplementary Fig. 3. ATF3 deficiency prevents steatosis-induced necroptosis after hepatectomy
Supplementary Fig. 4. Atf3 overexpression increases necroptosis in un-hepatectomised severe steatosis
Supplementary Fig. 5. eIF2α dephosphorylation and TNFα neutralisation in hepatic steatosis after hepatectomy
Supplementary Fig. 6. Schematic representation of the Ripk3 promoter region and the reporter plasmid
Supplementary Fig. 7. ATF3 switches apoptosis to necroptosis in hepatocytes
Supplementary Fig. 8. RIPK3 knockdown ameliorates NASH induced by MCD feeding
Supplementary Fig. 9. ATF3 knockout prevents MCD-induced NASH
Supplementary Fig. 10. Correlation of ATF3, RIPK3 or phosphorylated RIPK3 with blood aminotransferase levels in the liver of a patient with advanced NASH.
Supplementary References
Uncropped Gels and Blots for supplementary figures

Supplementary Methods

Analysis of plasma and hepatic parameters

Blood glucose levels were measured using a Glucocard G+ Meter (Arkray, Kyoto, Japan). Plasma aspartate aminotransferase/alanine aminotransferase (AST/ALT) levels were measured using the Transaminase CII-Test-Wako kit (Fujifilm Wako Pure Chemical Corp., Osaka, Japan). Plasma insulin concentrations were determined using a mouse insulin ELISA kit (Fujifilm Wako Pure Chemical Corp.). Hepatic triglyceride contents were measured using the triglyceride E-Test-Wako kit (Fujifilm Wako Pure Chemical Corp.)[1].

DNA methylation

We performed bisulfite pyrosequencing to quantitatively determine the methylation status of individual CpG sites[2]. Bisulfite treatment of 1 μg genomic DNA was performed using the EZ DNA Methylation-Gold kit (Zymo Research Inc., Seattle, WA) according to the manufacturer's instructions. Bisulfite-treated DNA was initially subjected to PCR amplification. The PyroMark PCR kit (Qiagen N.V., Venlo, Netherlands) was used to amplify the bisulfite-treated DNA according to the manufacturer's instructions. Pyrosequencing was performed using a PyroMark Q24 Pyrosequencer in conjunction with PyroMark Q24 Assay software 2.0.6 (Qiagen).

Separation of hepatic parenchymal and non-parenchymal cells

The liver was perfused using the following hepatocyte isolation method[3]. The liver of each anaesthetised mouse was perfused at a rate of 4.5 mL/min for the first 3 min with Hank's balanced salt solution (HBSS) (Fujifilm Wako Pure Chemical Corp.) containing 10 mM HEPES-NaOH (pH 7.4) and then for 15 min with HBSS containing collagenase type I (0.3 mg/ml) (Worthington, Lakewood, NY) and Protease Inhibitor Cocktail Complete-EDTA free (one tablet per 50 ml) (Roche, Basel, Switzerland). The perfused liver was harvested, incubated with HBSS(-) containing type I collagenase at 37°C for 15 min and mixed using GentleMACS Dissociators (Miltenyi Biotec, Bergisch Gladbach, Germany), first with the m_liver_03_01 dissociation program and then with the m_liver_04_01 program. All of the dissociated liver was filtered through a 100-μm filter membrane and the filtrate was centrifuged at 500 rpm for 10 min at 4°C. Percoll (Merck KGaA, Darmstadt, Germany) solutions (15%, 30% and 45%) were prepared by dilution with DMEM. The pellet was resuspended in 10 mL DMEM and gently laid on top of 10 mL of the 15% Percoll layer, 10 mL of the 30% Percoll layer and 15 mL of the 45% Percoll layer. Samples were centrifuged at 500 rpm for 10 min at 4°C in a swinging rotor. Parenchymal cells were collected from the pellets. Non-parenchymal cells were collected between the 15% and 30% Percoll layers.

Supplementary Table 1. Antibodies for immunoblotting and immunohistochemistry

Protein	Application	Species	Supplier	Catalogue No.	Concentration
Cleaved caspase-3	WB<br>IF	Mouse, Rat	Cell Signaling	#9661S	1:1000
Caspase-3	WB	Mouse, Rat	Cell Signaling	#9665	1:1000
Cleaved caspase-8	WB	Mouse	Cell Signaling	#8592S	1:1000
Caspase-8	WB	Mouse	ENZO	ALX-804-447	1:1000
p-RIPK3 (S232)	WB	Mouse	Abcam	ab195117	1:1000
RIPK3	WB	Mouse	Abcam	ab58828	1:1000
ACTIN	WB	Mouse, Rat	Sigma-Aldrich	A5441	1:2000
p-eIF2α (S51)	WB	Mouse	Cell Signaling	#3597S	1:1000
eIF2α	WB	Mouse	Cell Signaling	#9722	1:1000
CHOP	WB	Mouse	Santa Cruz	sc-575	1:200
ATF3	WB	Mouse	Santa Cruz	sc-188	1:200
p-MLKL (S345)	WB	Mouse	Abcam	ab196436	1:1000
MLKL	WB	Mouse	Cell Signaling	#28640	1:1000
Halo	WB		Promega	G9281	1:1000
GPX4	WB	Mouse	Abcam	ab125006	1:1000
GADD34	WB	Mouse	Santa Cruz	sc-825	1:200
p-c-Jun	WB	Mouse	Cell Signaling	#9261	1:1000
c-Jun	WB	Mouse	Santa Cruz	sc-1694	1:200
ATF3	IHC	Human	Abcam	Ab191513	1:200
RIPK3	IHC	Human	Cell Signaling	#10188	1:200
p-RIPK3	IHC	Human	Abcam	Ab209384	1:1000
GFP	WB<br>IHC		MBL	598	1:1000<br>1:100
mCherry	WB		Proteintech	26765-1-AP	1:1000
mCherry	IHC		SICGEN	AB0040-200	1:500
Cleaved caspase-8	WB	Rat	NOVUS	NB100-56116	1:1000
Caspase-8	WB	Rat	Proteintech	13423-1-AP	1:1000
p-RIPK3 (S316)	WB	Rat	Signalway Antibody	#12840	1:1000
RIPK3	WB	Rat	NOVUS	NBP1-77299	1:1000
p-MLKL (S358)	WB	Rat	Invitrogen	PA5-105678	1:1000
MLKL	WB	Rat	Proteintech	66675-1-Ig	1:1000

WB, western blot; IF, immunofluorescence; IHC, immunohistochemistry.

Supplementary Table 2. Specific primer sequences used for quantitative PCR

Gene	GenBank Accession No.	Sequence
Rplp0	NM_007475	Forward 5′-TTCCAGGCTTTGGGCATCA-3′<br>Reverse 5′-ATGTTCAGCATGTTCAGCAGTGTG-3′
Ripk3	NM_019955.2	Forward 5′-TTTACTGAGACTCCCGGTCCTCAC-3′<br>Reverse 5′-GTTCCCAATCTGCACTTCAGAACA-3′
Ripk1	NM_009068.3	Forward 5′-GCAGCATGACTGTGTACCCTTACC-3′<br>Reverse 5′-TGCGATCATTCTCGTCCTGTG-3′
Mlkl	NM_001310613.1	Forward 5′-TGCTGCTTCAGGTTTATCATTGG-3′<br>Reverse 5′-CACGCTAATTTGCAACTGCATC-3′
Tnf	NM_013693.2	Forward 5′-AAGCCTGTAGCCCACGTCGTA-3′<br>Reverse 5′-GGCACCACTAGTTGGTTGTCTTTG-3′
Gpx4	NM_001037741.4	Forward 5′-TGCAACAGCTCCGAGTTCCT-3′<br>Reverse 5′-GTGACGATGCACACGAAACC-3′
Slc7a11	NM_011990.2	Forward 5′-AACGCCCAGATATGCATCGTC-3′<br>Reverse 5′-GGTGCTGAATGGGTCCGAGTA-3′
Ddit3	NM_007837.3	Forward 5′-AATAACAGCCGGAACCTGAGGA-3′<br>Reverse 5′-ACTCAGCTGCCATGACTGCAC-3′
Atf3	NM_007498.3	Forward 5′-CGAAGACTGGAGCAAAATG-3′<br>Reverse 5′-AGGTTAGCAAAATCCTCAAATAC-3′
Tgfb1	NM_011577.1	Forward 5′-TACGGCAGTGGCTGAACCAA-3′<br>Reverse 5′-CGGTTCATGTCATGGATGGTG-3′
Acta2	NM_007392.3	Forward 5′-GACAATGGCTCTGGGCTCTGTA-3′<br>Reverse 5′-TTTGGCCCATTCCAACCATTA-3′
Col1a1	NM_007742.3	Forward 5′-GACATGTTCAGCTTTGTGGACCTC-3′<br>Reverse 5′-GGGACCCTTAGGCCATTGTGTA-3′
rAtf3	NM_012912.2	Forward 5′-GCTGCTGCCAAGTGTCGAA-3′<br>Reverse 5′-CGGTGCAGGTTGAGCATGTA-3′
rRipk3	NM_139342.1	Forward 5′-CTACTGCACCGGGACCTCAA-3′<br>Reverse 5′-GTGGACAGGCCAAAGTCTGCTA-3′
rMlkl	XM_008772571.2	Forward 5′-TGGAAAGATCCCGTTTGAAG-3′<br>Reverse 5′-TGATCTCCTGCAACAACTCG-3′
Gapdh	NM_017008.4	Forward 5′ -GGCACAGTCAAGGCTGAGAATG-3′<br>Reverse 5′ -ATGGTGGTGAAGACGCCAGTA-3′
Alb	NM_009654.4	Forward 5′ -TGTGCAGAGGCTGACAAGGA-3′<br>Reverse 5′ -TCTGACGGACAGATGAGACCAA-3′
Adgre1	NM_010130.4	Forward 5′ -AGCTGTAACCGGATGGCAAAC-3′<br>Reverse 5′ -GACTGTACCCACATGGCTGATGA-3′
Cdh5	NM_009868.4	Forward 5′ -TGCAACAGACAAGGATGTGGTG-3′<br>Reverse 5′ -ACTTGGCATGCTCCCGATTA-3′
Des	NM_010043.2	Forward 5′ -GACGACCTGCAGAGGCTCAA-3′<br>Reverse 5′ -GATTCTGCGCTCCAGGTCAA-3′
Gfp	LT726937.1	Forward 5′ -GAGCGCACCATCTTCTTCAAG-3′<br>Reverse 5′ -AGCTTGTGCCCCAGGATGTTG-3′
mCherry	MW247150.1	Forward 5′ -CCCCGTAATGCAGAAGAAGA-3′<br>Reverse 5′ -GCTTCTTGGCCTTGTAGGTG-3′

Supplementary Table 3. Sequences of various lengths and their mutants used to construct the Ripk3 promoter

Plasmid	Sp1 Binding Site	Sequence
pGL4.27_mRipk3 promoter_−504/+210	GCTCCACCCCA (WT)	Forward 5′-TATAGGTACCACCCTGTCTCAAAAGTAAGT-3′<br>Reverse 5′-TATAAAGCTTAGCGGCTTCCTGGAGTTATG-3′
pGL4.27_mRipk3promoter_−255/+210	GCTCCACCCCA (WT)	Forward 5′-TATAGGTACCCTCCGTGATCTTAGGACC-3′<br>Reverse 5′-TATAAAGCTTAGCGGCTTCCTGGAGTTATG-3′
pGL4.27_mRipk3promoter_−140/+210	GCTCCACCCCA (WT)	Forward 5′-TATAGGTACCAATCGTTCCTGGATGGTGAG-3′<br>Reverse 5′-TATAAAGCTTAGCGGCTTCCTGGAGTTATG-3′
pGL4.27_mRipk3promoter_−60/+210	GCTCCACCCCA (WT)	Forward 5′-TATAGGTACCACGTGCACAGGAAATAGC-3′<br>Reverse 5′-TATAAAGCTTAGCGGCTTCCTGGAGTTATG-3′
pGL4.27_mRipk3promoter_−12/+210	None	Forward 5′-TATAGGTACCGCGGAGATAGAAGGAGCCTC-3′<br>Reverse 5′-TATAAAGCTTAGCGGCTTCCTGGAGTTATG-3′
pGL4.27_mRipk3 promoter_−504/+210: Sp1-mt1	GCTCCA***G***CCCA (C→G)	Forward 5′-GTTTTCAATGCTCCAGCCCAGGGGGCGGAG-3′<br>Reverse 5′-CTCCGCCCCCTGGGCTGGAGCATTGAAAAC-3′
pGL4.27_mRipk3 promoter_−504/+210: Sp1-mt2	GCTC***G***ACCCCA (C→G)	Forward 5′-GTTTTCAATGCTCGACCCCAGGGGGCGGAG-3′<br>Reverse 5′-CTCCGCCCCCTGGGGTCGAGCATTGAAAAC-3′
pGL4.27_mRipk3 promoter_−504/+210: Sp1-mt3	GCTC***G***A***G***CCCA (C→G, C→G)	Forward 5′-GTTTTCAATGCTCGAGCCCAGGGGGCGGAG-3′<br>Reverse 5′-CTCCGCCCCCTGGGCTCGAGCATTGAAAAC-3′

Supplementary Table 4. Antisense probe sequences for in situ hybridisation

1) Mouse activating transcription factor 3 (Atf3)<br>Accession #: NM_007498.3
CTCAGAATGGACGGACACCGGAAGACGAGAGGAACCTCTTTATCCAACAGATAAAAGAAGGAA<br>CATTGCAGAGCTAAGCAGAGGTGGCACGGAGGCAATTGGGGAGTTCTTACTGAATCCTCCTTTT<br>CCACCCCACACCCTGAAGCCATTGGAAAACTGGCTTCCTGTGCACTTCTAGAATCCCAGCAGCC<br>AAGAGCCGTTGGGGCAGGAGGGCCTGTGGTGACCTACTGCATTGACCCACTCTGCCCCCGAGTG<br>AACCGTGGAGCAGGCAGGAGCATCCTTTGTCTCACCAATTCCAGGATTTAGGCCTTATCATCCC<br>GGCCAGTCTCAGATGACCTAGCTGGCCCCAGGCTGGGGTCCTATGCAAAGCAGGATCCCACTAA<br>TGGGATTCAGGCAGAAGTGTCTACCTTGATAGGTGGGGTGGGACCACATCCTCCACTGTGGCTG<br>ACAACGCCCTTCCAAGGGAATATGGAATGAGAACATTCATTATTGAGGTTGTCCAATGGCCAGG<br>GTATGCTTTCTAGAAAATATGCTGTTCTGTCCCAGAATGACTGTGCATAGGGTATCCGTTTCAGA<br>GCCTGGTGTTGTGCTATTTAGATGTTTGTCTTGCACAACATTGGCATGATTTTTCCGGGAGTTTC<br>ATCAGATCTGATTTCTGAGAGTCTGGGGATCTGCCATGGTGGAAAGTGCCCCTCAAAAGCATTT<br>GTGTGGCCACATGAACTGGCTGGCACCAGGGGAGTGAAACTGGCTGATGACCAGCTGAGCCAC<br>TTTGTGCCAACAGAGGATGGACGACACCTTTCCCTGTACCCACTGCAGAGGAAGAACCCTGGGC<br>ACAGCAGCTTTGTCCTTGGCTACAAACTGTTACAACGTCACACAATGAAGGCACAAAGTCCAAC<br>TTTCAAAGGGTGTAGGACTCCATACTCAGTGACAGGGCAGGAAGAGCCAAAGATAACCACAGC
2) Mouse receptor-interacting serine-threonine kinase 3 (Ripk3)<br>Accession #: NM_019955.2
GAATTCGGGGCCTGAACCTCCGTGCCTTGACCTACTGATTGTTCCCTTTGCAGACCCTGCCTTCC<br>TCTCAGTCCACACTCCGAGCCGTAGACGTCGGGTTTCCAGCTCCGTCTGTGCACACATAACTCCA<br>GGAAGCCGCTAGCTCCCGACGATGTCTTCTGTCAAGTTATGGCCTACTGGTGCGTCAGCGGTTCC<br>TCTGGTGAGCCGTGAAGAACTGAAGAAGCTGGAGTTTGTGGGTAAAGGAGGGTTCGGAGTCGT<br>GTTCCGGGCACACCACAGAACATGGAACCATGATGTAGCAGTCAAGATCGTGAACTCGAAGAA<br>GATATCCTGGGAGGTGAAGGCTATGGTTAATCTTCGTAATGAGAACGTTCTGCTCCTGCTGGGG<br>GTCACTGAGGACCTCCAGTGGGACTTCGTGTCCGGGCAGGCTCTGGTGACAAGATTCATGGAGA<br>ATGGCTCCCTCGCAGGGCTGCTGCAACCCGAGTGCCCTCGGCCCTGGCCACTCCTCTGTCGCCTG<br>CTGCAGGAAGTGGTGCTGGGGATGTG

Supplementary Table 5. Baseline characteristics by stage

	Stage 0	Stage 1–2	Stage 3–4	Total	p*
Age at biopsy (years)	50.3 ± 3.8 (*n* = 3)	47.9 ± 4.7 (*n* = 16)	63.5 ± 2.6 (*n* = 29)	57.1 ± 2.5 (*n* = 43)	-
ALT (U/L)	89.0 ± 38.0 (*n* = 2)	123.7 ± 25.9 (*n* = 10)	106.7 ± 22.3 (*n* = 13)	112.1 ± 15.5 (*n* = 25)	-
AST (U/L)	48.5 ± 12.5 (*n* = 2)	66.1 ± 10.8 (*n* = 10)	90.2 ± 11.5 (*n* = 13)	77.2 ± 7.8 (*n* = 25)	-

*p-values derived from one-way ANOVA followed by Tukey–Kramer's honestly significant difference test. Data are presented as the mean values ± SEM. Only laboratory values from patients who underwent liver biopsy were included. ALT, alanine aminotransferase; AST, aspartate aminotransferase.

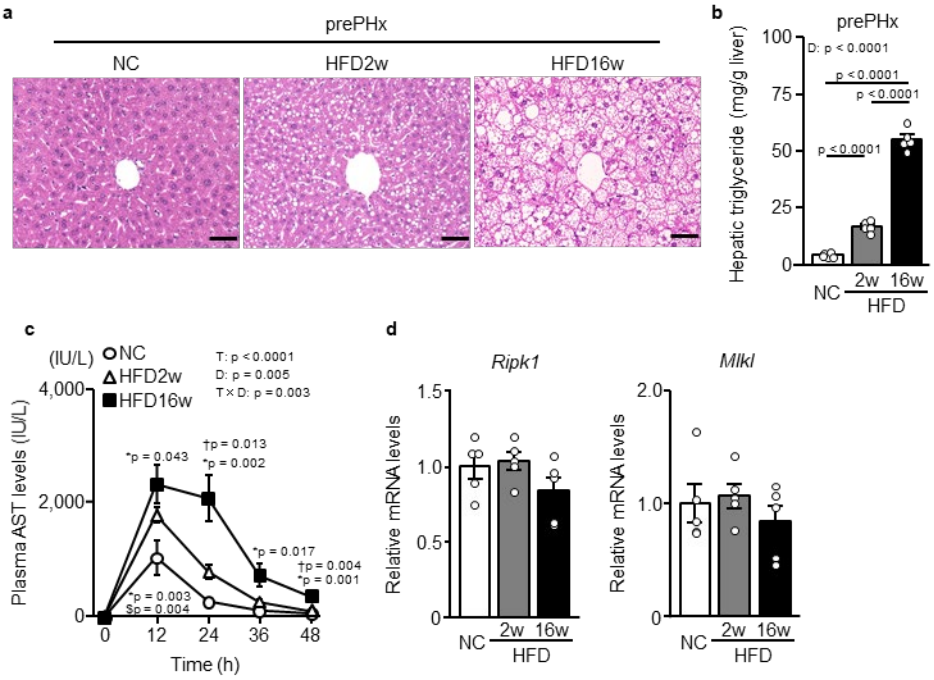

Supplementary Fig. 1. Non-apoptotic cell death is increased in severe steatosis after hepatectomy

(a–b) Severity of hepatic steatosis in mice fed normal chow (NC) or a HFD for 2 or 16 weeks. (a) Haematoxylin-eosin staining of liver samples collected pre-hepatectomy (prePHx). Scale bar, 50 µm. (b) Hepatic triglyceride levels. (c–d) Mice fed NC or a HFD for 2 or 16 weeks underwent 70% partial hepatectomy. (c) Plasma aspartate aminotransferase (AST) levels over time after hepatectomy. (d) Quantitative PCR analysis. Data are presented as the mean values ± SEM. [*n* = 5/group, biologically independent samples]. Statistics: one-way ANOVA followed by Tukey's multiple comparisons test (b, d), one-way repeated-measures ANOVA followed by Bonferroni's multiple comparisons test (c).* indicates p-values for HFD16w versus NC; † indicates p-values for HFD16w versus HF2w; $ indicates p-values for HFD2w versus NC; T, time effect; D, diet effect; T×D, time and diet interaction. Source data are provided as a Source Data file.

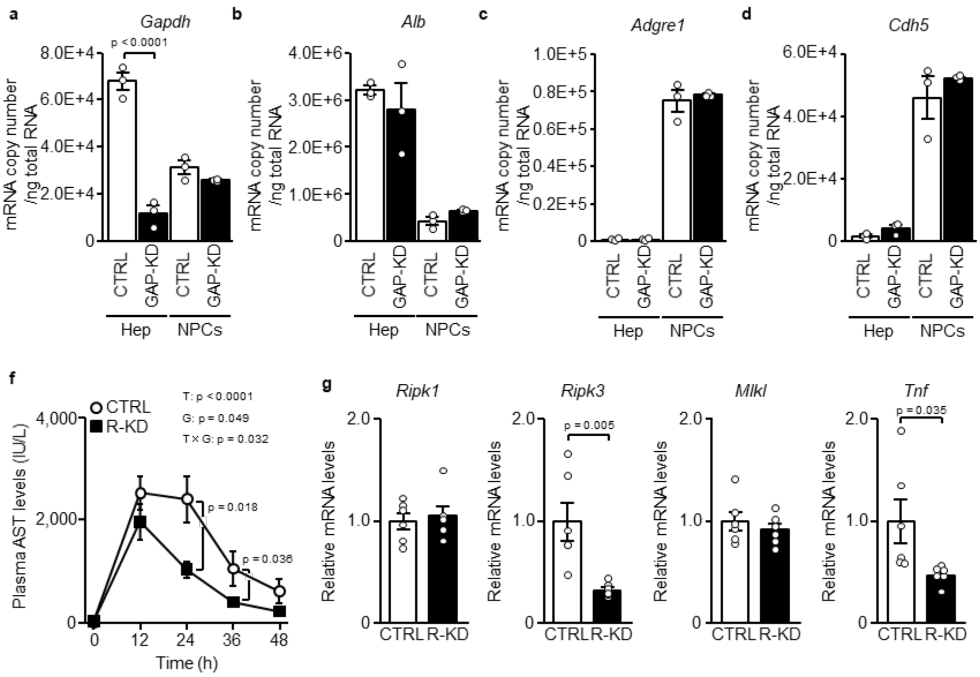

Supplementary Fig. 2. Non-apoptotic cell death due to necroptosis in severe steatosis after hepatectomy

(a–e) Hepatic cellular specificity of knockdown by Gapdh siRNA. (f–g) HFD-fed mice received an intravenous injection of Ripk3 or control siRNA and underwent 70% partial hepatectomy. (f) Plasma AST levels. (g) Quantitative PCR analysis. Data are presented as the mean values ± SEM. [(a–e) n = 3/group; (f–g) n = 6/group, biologically independent samples]. Statistics: two-way ANOVA followed by Bonferroni's multiple comparisons test (a–e), one-way repeated-measures ANOVA followed by Bonferroni's multiple comparisons test (f), two-tailed Student's t-test (g). Hep, Hepatocyte; NPCs, hepatic non-parenchymal cells; CTRL, control group; GAP-KD, Gapdh knockdown group; T, time effect; G, group effect; T×G, time and group interaction; CTRL, control group; R-KD, Ripk3 knockdown group. Source data are provided as a Source Data file.

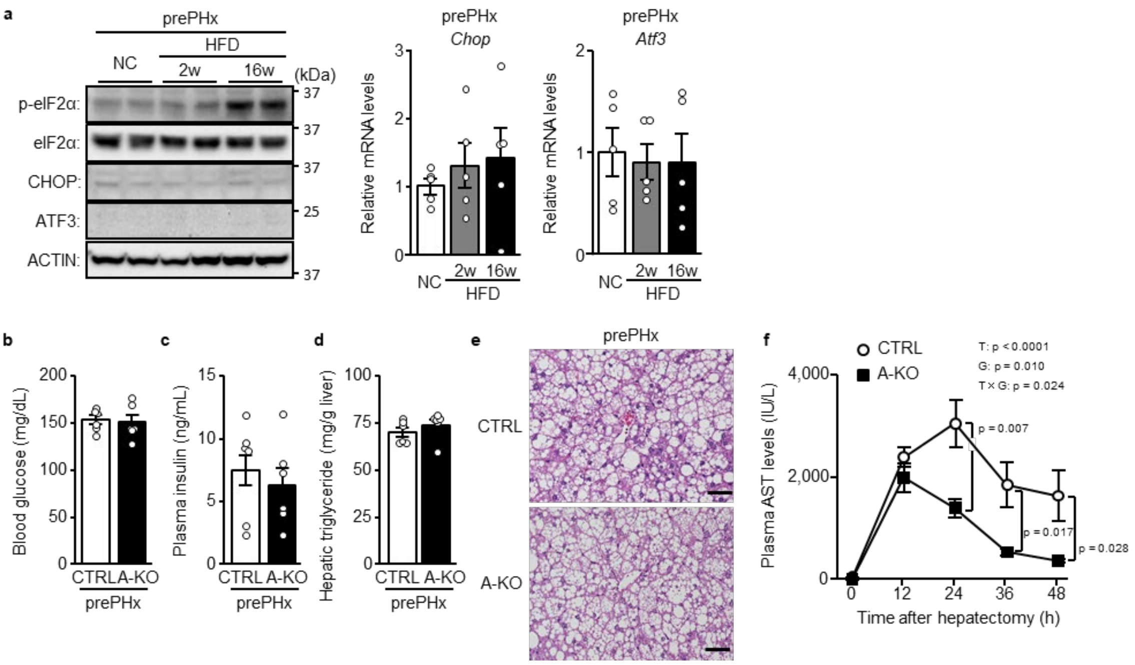

Supplementary Fig. 3. ATF3 deficiency prevents steatosis-induced necroptosis after hepatectomy

(a) Mice were fed normal chow (NC) or a HFD for 2 or 16 weeks pre-hepatectomy (prePHx). Immunoblot analysis (left). Quantitative PCR analysis (right). (b–f) A-KO mice and littermates (CTRL) were fed a HFD for 16 weeks. (b–e) Pre-hepatectomy (prePHx) data. (b) Blood glucose levels. (c) Plasma insulin levels. (d) Hepatic triglyceride levels. (e) Haematoxylin-eosin staining of liver samples. Scale bar, 50 µm. (f) Plasma AST levels over time after hepatectomy. Data are presented as the mean values ± SEM. [(a) *n* = 5/group; (b–f) *n* = 6/group, biologically independent samples]. Statistics: one-way ANOVA followed by Tukey's multiple comparisons test (a), two-tailed Student's *t*-test (b–d), one-way repeated-measures ANOVA followed by Bonferroni's multiple comparisons test (f). T, time effect; G, group effect; T×G, time and group interaction. Source data are provided as a Source Data file.

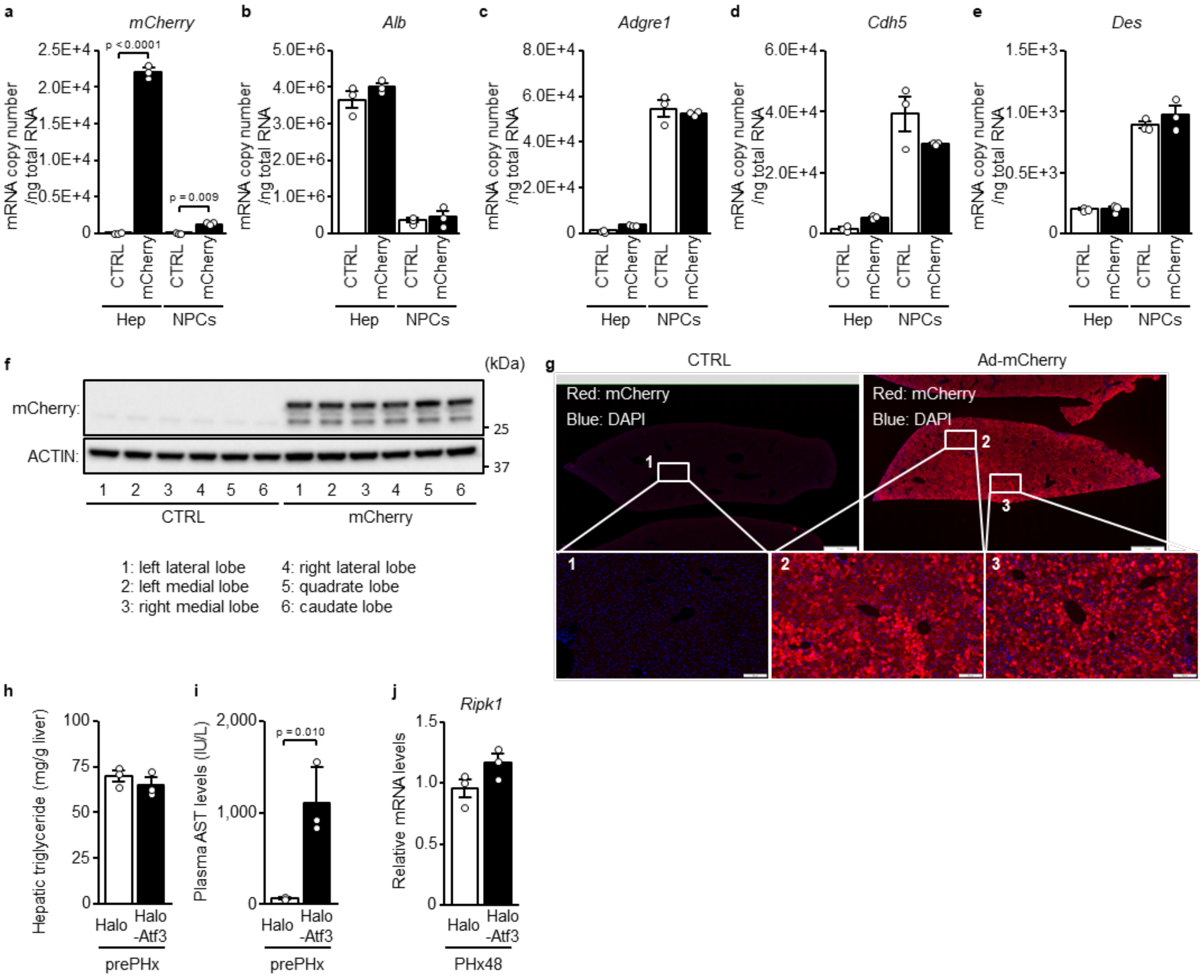

Supplementary Fig. 4. Atf3 overexpression increases necroptosis in un-hepatectomised severe steatosis

(a–g) Mice were infected with adenovirus encoding mCherry. mCherry expression was hepatocyte-dominant (a–e) and ubiquitously expressed throughout the liver (f–g). Scale bar, 1 mm ((g) top). Scale bar, 100 µm ((g) bottom). (h–j) Wild-type mice were fed a HFD for 16 weeks and halo-Atf3 or halo, as control, was overexpressed by adenovirus infection. (h) Hepatic triglyceride levels. (i) Plasma AST levels. (j) Quantitative PCR analysis of *Ripk1*. Data are presented as the mean values ± SEM. [n = 3/group, biologically independent samples]. Statistics: two-way ANOVA followed by Bonferroni's multiple comparisons test (a–e), two-tailed Student's t-test (h–j). Source Data are provided as a Source Data file.

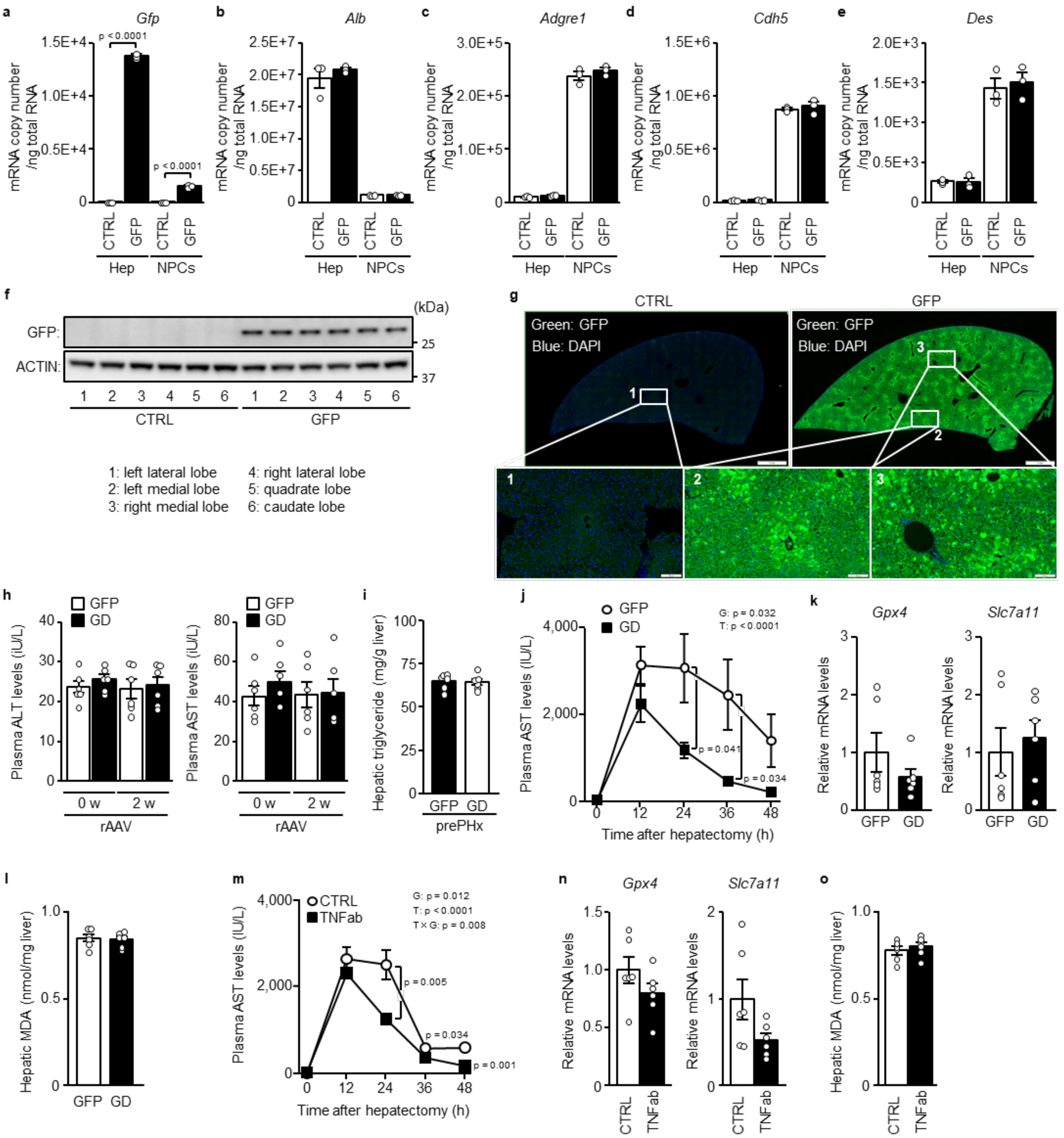

Supplementary Fig. 5. eIF2α dephosphorylation and TNFα neutralisation in hepatic steatosis after hepatectomy

(a–g) Mice were infected with recombinant adeno-associate virus (rAAV) encoding GFP. GFP expression was hepatocyte-dominant (a–e) and ubiquitously expressed throughout the liver (f–g). Scale bar, 1 mm ((g) top). Scale bar, 100 μm ((g) bottom). (h–l) Mice with severe hepatic steatosis overexpressing Gadd34 (GD) or GFP by rAAV underwent partial hepatectomy 2 weeks after rAAV infection. (h) Plasma ALT and AST levels before and after 2 weeks infection (0w and 2w, respectively). (i) Hepatic triglyceride levels pre-hepatectomy (prePHx). (j) Plasma AST levels after hepatectomy. (k) Quantitative PCR analysis of genes

related to ferroptosis. (l) Hepatic MDA levels. (m–o) Mice with severe hepatic steatosis underwent partial hepatectomy and were injected with TNFα-neutralising antibody (TNFab) or anti-IgG (CTRL) 12 hours after hepatectomy. (m) Plasma AST levels. (n) Quantitative PCR analysis of genes related to ferroptosis. (o) Hepatic MDA levels. Data are presented as the mean values ± SEM. [(a–f) n = 3/group; (h–o) n = 6/group, biologically independent samples]. Statistics: two-way ANOVA followed by Bonferroni's multiple comparisons test (a–e), two-tailed Student's t-test (h, i, k, l, n, o), one-way repeated-measures ANOVA followed by Bonferroni's multiple comparisons test (j, m). T, time effect; G, group effect; T×G, time and group interaction. Source data are provided as a Source Data file.

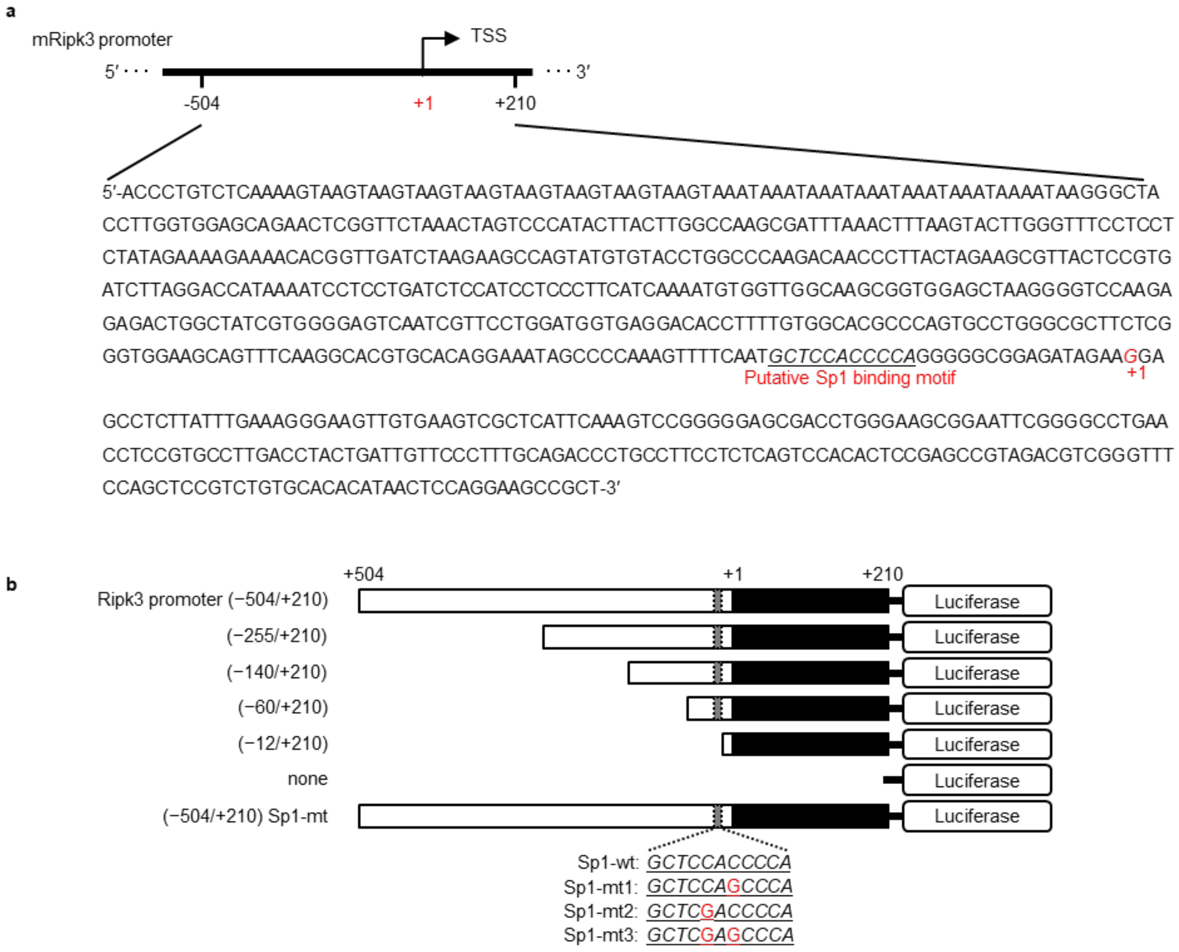

Supplementary Fig. 6. Schematic representation of the Ripk3 promoter region and the reporter plasmid

(a) Sequence of the Ripk3 promoter region from −504 to +210. The underlined region indicates the putative Sp1 binding motif (−27/−17). (b) Schema of reporter plasmids of various lengths and their mutants (mt) for the Ripk3 promoter of the putative Sp1 binding motif.

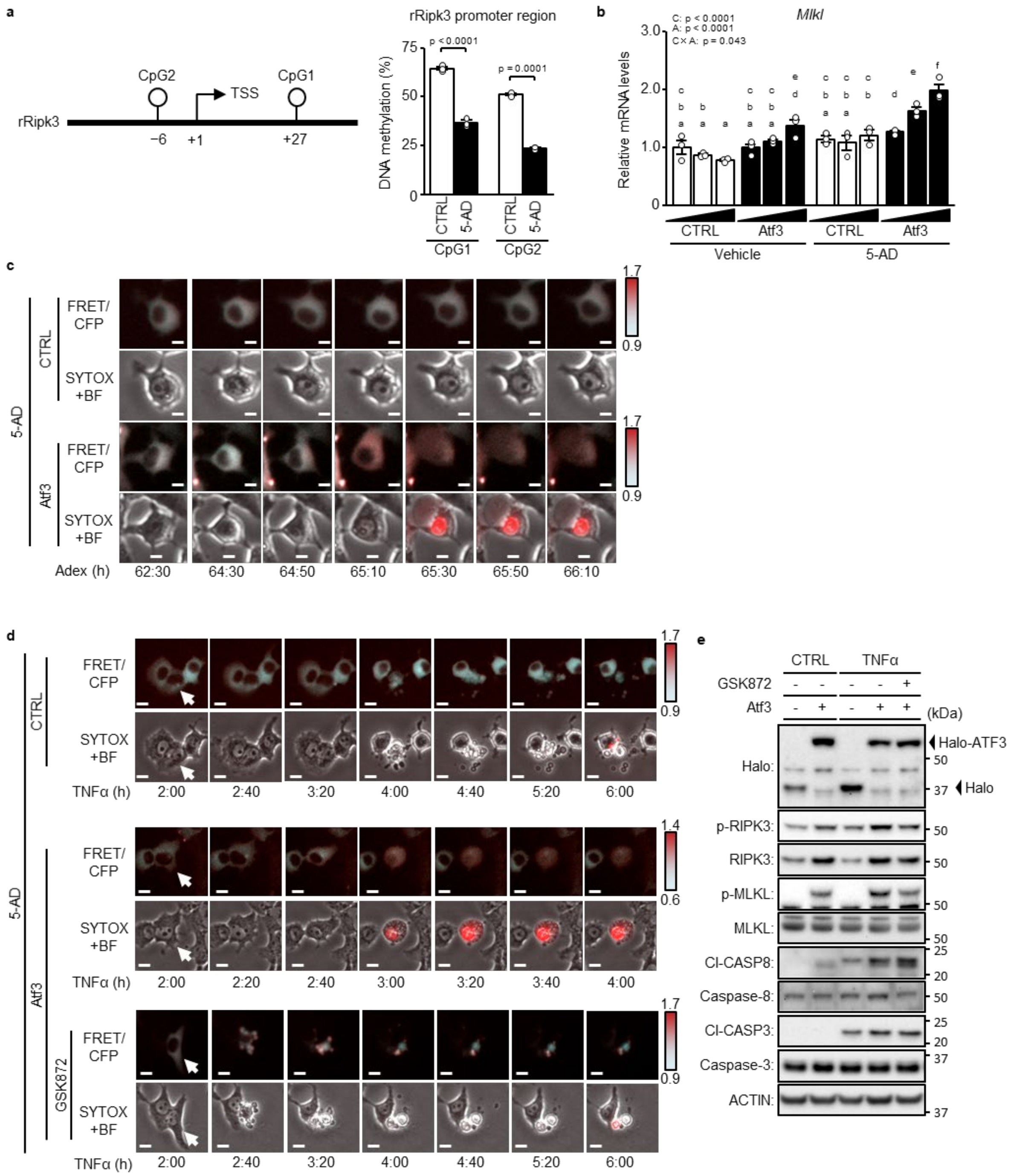

Supplementary Fig. 7. ATF3 switches apoptosis to necroptosis in hepatocytes

(a) Schema of CpG in the Ripk3 promoter region of rat (left) and the DNA methylation levels of CpG (right). (b) Halo-Atf3 or halo was overexpressed by adenovirus in rat H4IIE cells treated with/without 5-AD. Expression levels of Mlkl by quantitative PCR. (c) Time-lapse images of a single cell. Halo-Atf3 or halo was overexpressed by adenovirus in H4IIE-SMART cells treated with 5-AD. Scale bar, 5 µm. (d) Time-lapse images of a single cell. Halo-Atf3 or halo was overexpressed by adenovirus in H4IIE-SMART cells treated with 5-AD. H4IIE-SMART cells were stimulated with TNFα and GSK872. Scale bar, 5 µm. (e)

Immunoblot analysis. H4IIE cells were stimulated with TNFα and GSK872. Data are presented as the mean values ± SEM. [n = 3/group, biologically independent samples]. Statistics: two-tailed Student's t-test (a), two-way ANOVA followed by Tukey's multiple comparisons test (b). C, chemical effect; A, adenovirus effect; C×A, chemical and adenovirus interaction. Source data are provided as a Source Data file.

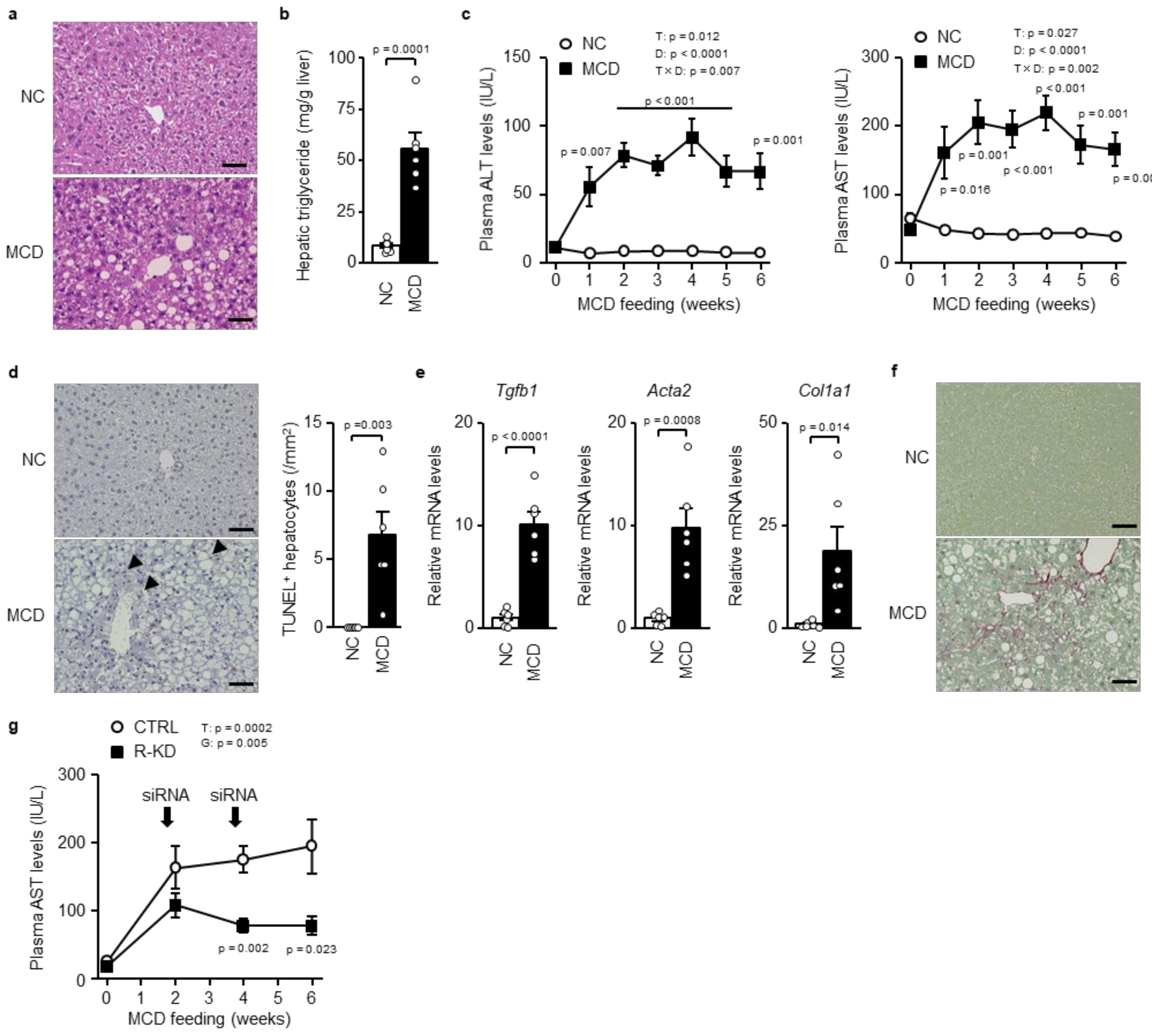

Supplementary Fig. 8. RIPK3 knockdown ameliorates NASH induced by MCD feeding
(a–f) Wild-type mice were fed an MCD for 6 weeks. (a) Haematoxylin-eosin staining of liver samples. Scale bar, 50 µm. (b) Hepatic triglyceride levels. (c) Plasma ALT and AST levels during MCD feeding. (d) TUNEL staining (left). Scale bar, 50 µm. Arrowheads indicate TUNEL$^+$ hepatocytes. Number of TUNEL$^+$ hepatocytes (right). (e) Quantitative PCR analysis. (f) Sirius red staining. Scale bar, 50 µm. (g) Ripk3 siRNA was injected into the mice at 2 and 4 weeks after MCD feeding. Plasma AST levels during MCD feeding. Data are presented as the mean values ± SEM. [n = 6/group, biologically independent samples]. Statistics: two-tailed Student's t-test (b, d, e), one-way repeated-measures ANOVA followed by Bonferroni's multiple comparisons test (c, g). R-KD, Ripk3 knockdown; T, time effect; D, diet effect; T×D, time and diet interaction; G, group effect. Source data are provided as a Source Data file.

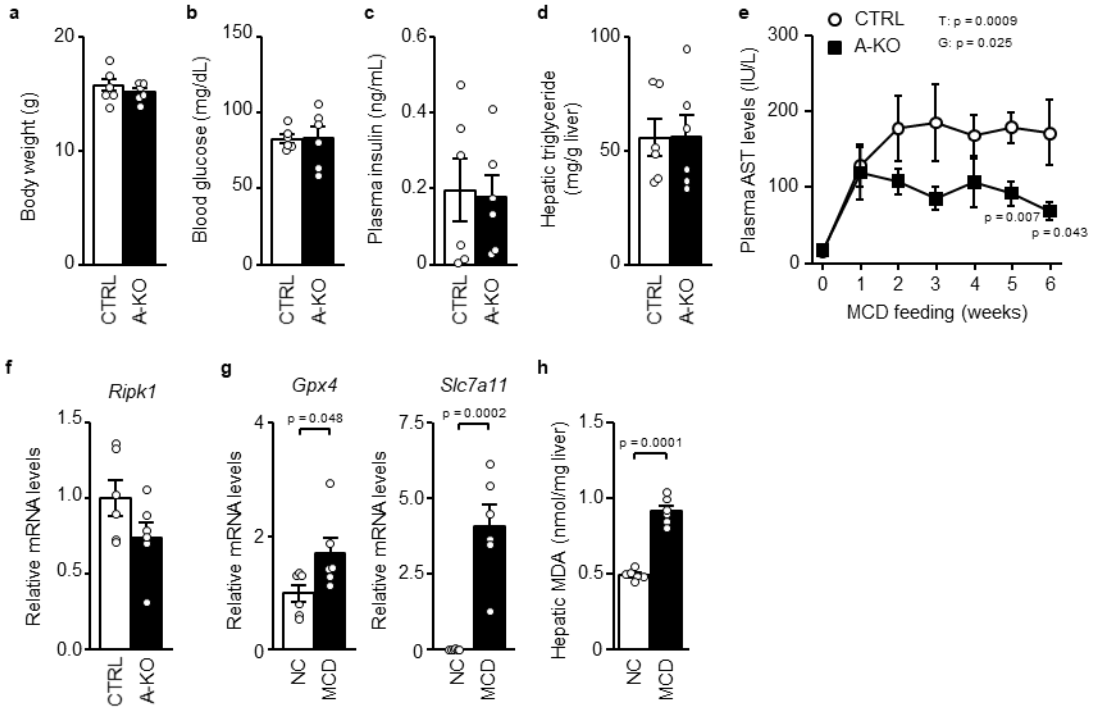

Supplementary Fig. 9. ATF3 knockout prevents MCD-induced NASH

(a–f) ATF3 knockout mice (A-KO) and littermates (CTRL) were fed an MCD for 6 weeks. (a) Body weight. (b) Blood glucose concentration. (c) Plasma insulin levels. (d) Hepatic triglyceride levels. (e) Plasma AST levels at the indicated time points. (f) Quantitative PCR analysis of *Ripk1*. (g–h) Wild-type mice were fed an MCD for 6 weeks. (g) Quantitative PCR analysis of genes related to ferroptosis. (h) Hepatic MDA levels. Data are presented as the mean values ± SEM. [n = 6/group, biologically independent samples]. Statistics: two-tailed Student's t-test (a–d, f–h), one-way repeated-measures ANOVA followed by Bonferroni's multiple comparisons test (e). T, time effect; G, group effect. Source data are provided as a Source Data file.

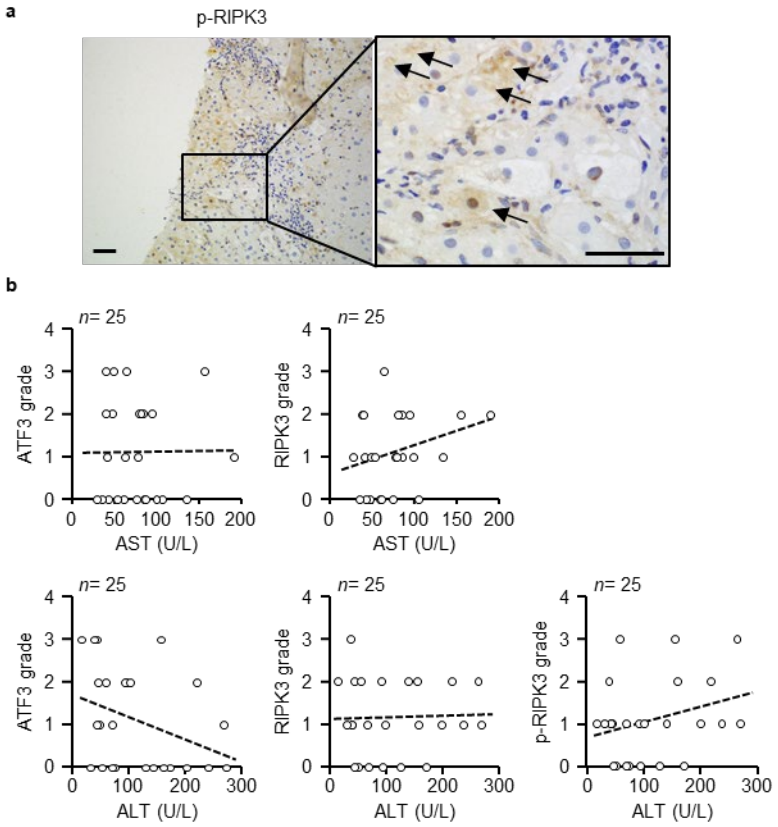

Supplementary Fig. 10. Correlation of ATF3, RIPK3 or phosphorylated RIPK3 with blood aminotransferase levels in the liver of a patient with advanced NASH.

(a) Images of phosphorylated RIPK3 (p-RIPK3) staining. Scale bar, 50 µm. (b) Correlation of ATF3, RIPK3 or p-RIPK3 with blood aminotransferase levels, calculated using Spearman's rank correlation test. The liver biopsy samples are biologically independent samples. Source data are provided as a Source Data file.

Supplementary References

1. Hashiuchi, E., *et al.* Diet Intake Control Is Indispensable for the Gluconeogenic Response to Sodium-Glucose Cotransporter 2 Inhibition in Male Mice. *J Diabetes Investig.* **12**, 35-47 (2021).
2. Hori, N., *et al.* Aberrant CpG methylation of the imprinting control region KvDMR1 detected in assisted reproductive technology-produced calves and pathogenesis of large offspring syndrome. *Anim Reprod Sci* **122**, 303-312 (2010).
3. Inaba, Y., et al. Growth arrest and DNA damage-inducible 34 regulates liver regeneration in hepatic steatosis in mice. Hepatology 61, 1343-1356 (2015).

Uncropped Gels and Blots

Uncropped images of original representative immunoblotting scans.

in Supplementary Figure 3a

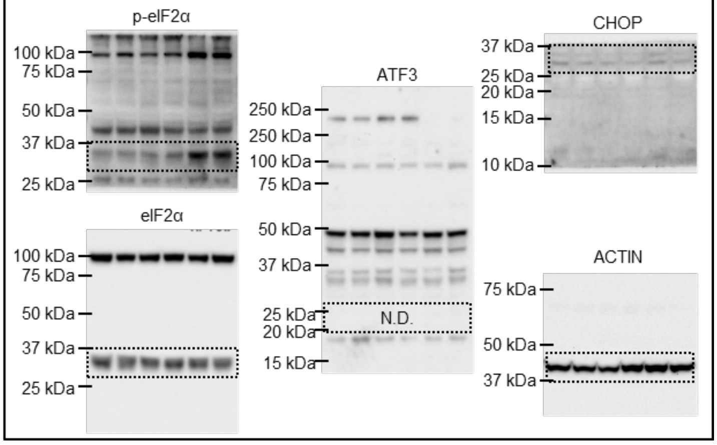

in Supplementary Figure 4f

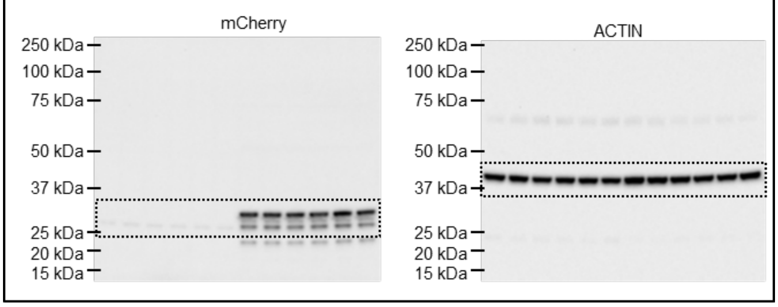

in Supplementary Figure 5f

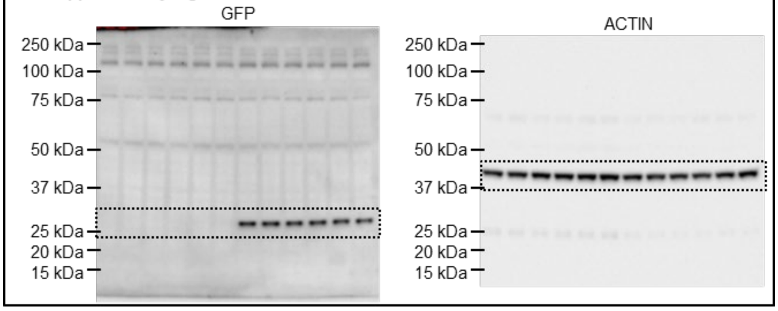

in Supplementary Figure 7e

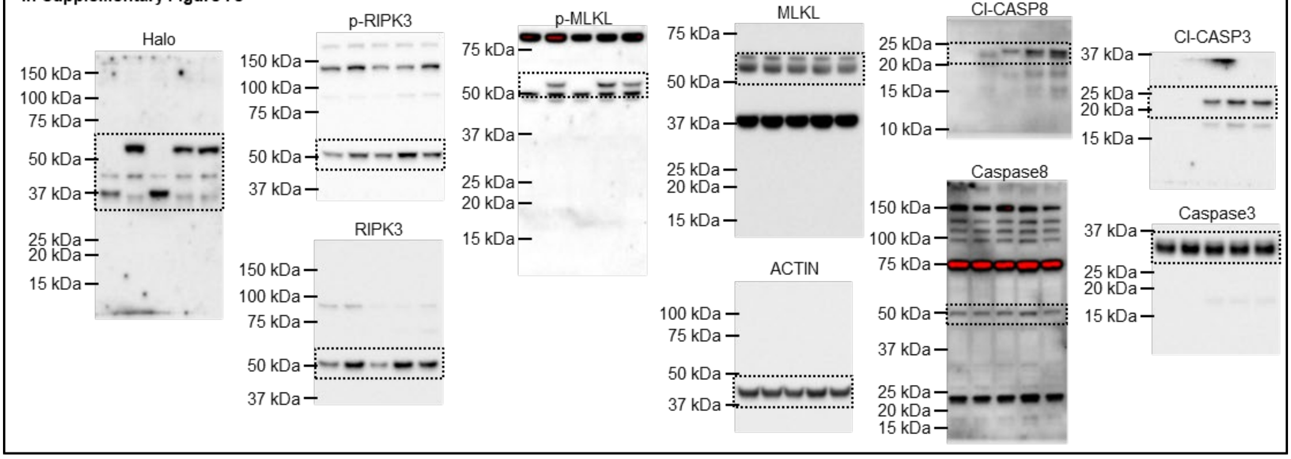

Uncropped images of haematoxylin and eosin, TUNEL and Sirius red staining.

in Supplementary Figure 1a

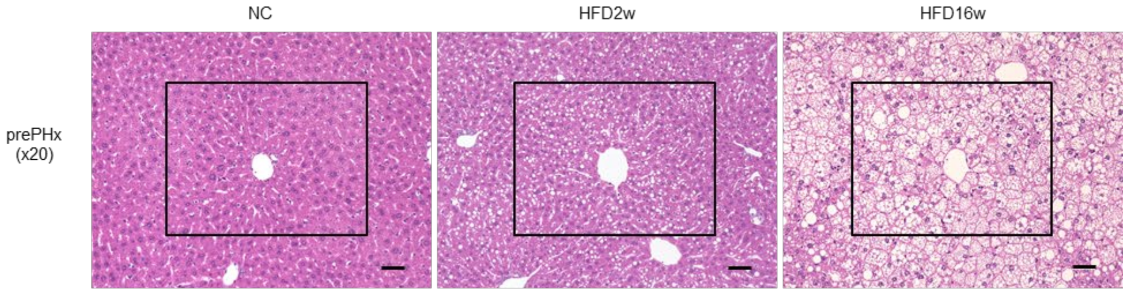

in Supplementary Figure 3e

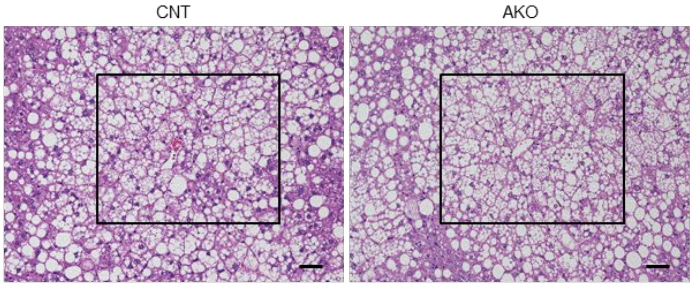

in Supplementary Figure 8a

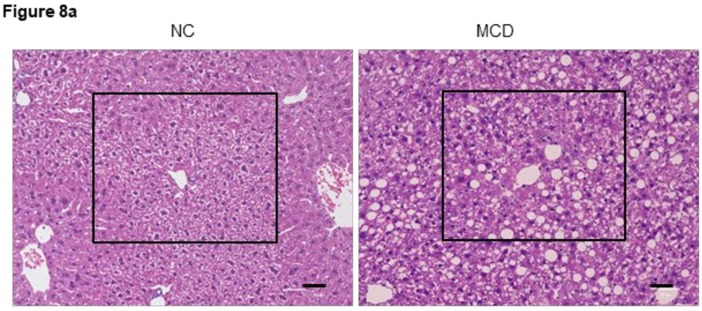

in Supplementary Figure 8d

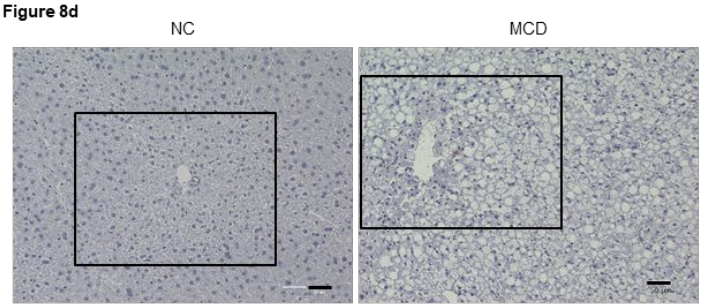

in Supplementary Figure 8f

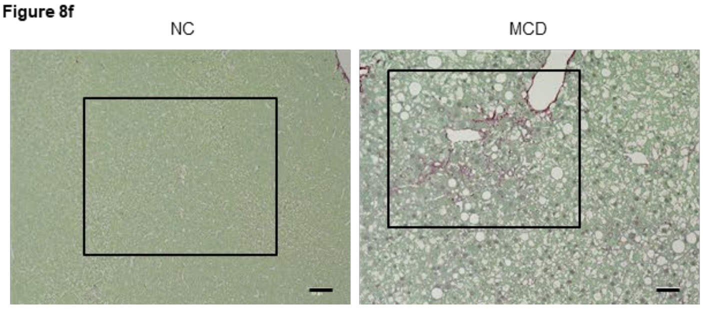

Supplement: Supplementary file 1 — Supplementary Information [file 41467_2023_35804_MOESM1_ESM.pdf]
