# Supplementary material for: The transcription factor ATF3 switches cell death from apoptosis to necroptosis in hepatic steatosis in male mice
Source: Nat Commun. 2023 Jan 23;14:167. doi: 10.1038/s41467-023-35804-w (PMC9871012; doi:10.1038/s41467-023-35804-w)
Supplement: Supplementary file 3 — Description of Additional Supplementary Files [file 41467_2023_35804_MOESM3_ESM.pdf]

Description of Additional Supplementary Files

File Name: Supplementary Movie 1
Description: (a) Live imaging of Fig. 7c. Halo-Atf3 (Atf3) or halo (CTRL) was overexpressed by adenovirus in H4IIE-SMART cells treated with/without 5-AD (3 μM). Cell death was monitored by SYTOX and necroptosis by FRET. The time shown in the figure is the time after virus infection. BF, bright-field. (b) Live imaging of Supplementary Fig. 7c. Halo-Atf3 (Atf3) or halo (CTRL) was overexpressed by adenovirus in H4IIE-SMART cells treated with/without 5-AD (3 μM). Cell death was monitored by SYTOX and necroptosis by FRET. The time shown in the figure is the time after virus infection. BF, bright-field.

File Name: Supplementary Movie 2
Description: (a) Live imaging of Fig. 7g, i and k. Halo-Atf3 (Atf3) or halo (CTRL) was overexpressed by adenovirus in H4IIE-SMART cells treated with 5-AD (3 μM). GSK872 (5 μM) was added 24 h after infection. TNFα (25 ng/mL) and SYTOX (1 μM) were added 41 h after infection. Cell death was monitored by SYTOX and necroptosis by FRET/CFP. The time shown in the figure is the time after treatment with TNFα. BF, bright-field. (b) Live imaging of Supplementary Fig. 7d. Halo-Atf3 (Atf3) or halo (CTRL) was overexpressed by adenovirus in H4IIE-SMART cells treated with 5-AD (3 μM). GSK872 (5 μM) was added 24 h after infection. TNFα (25 ng/mL) and SYTOX (1 μM) were added 41 h after infection. Cell death was monitored by SYTOX and necroptosis by FRET/CFP. The time shown in the figure is the time after treatment with TNFα. BF, bright-field.
