# Supplementary material for: The transcription factor ATF3 switches cell death from apoptosis to necroptosis in hepatic steatosis in male mice
Source: Nat Commun. 2023 Jan 23;14:167. doi: 10.1038/s41467-023-35804-w (PMC9871012; doi:10.1038/s41467-023-35804-w)
Supplement: Supplementary file 4 — Supplementary Movie 1 [file 41467_2023_35804_MOESM4_ESM.pptx]

## Slide 1
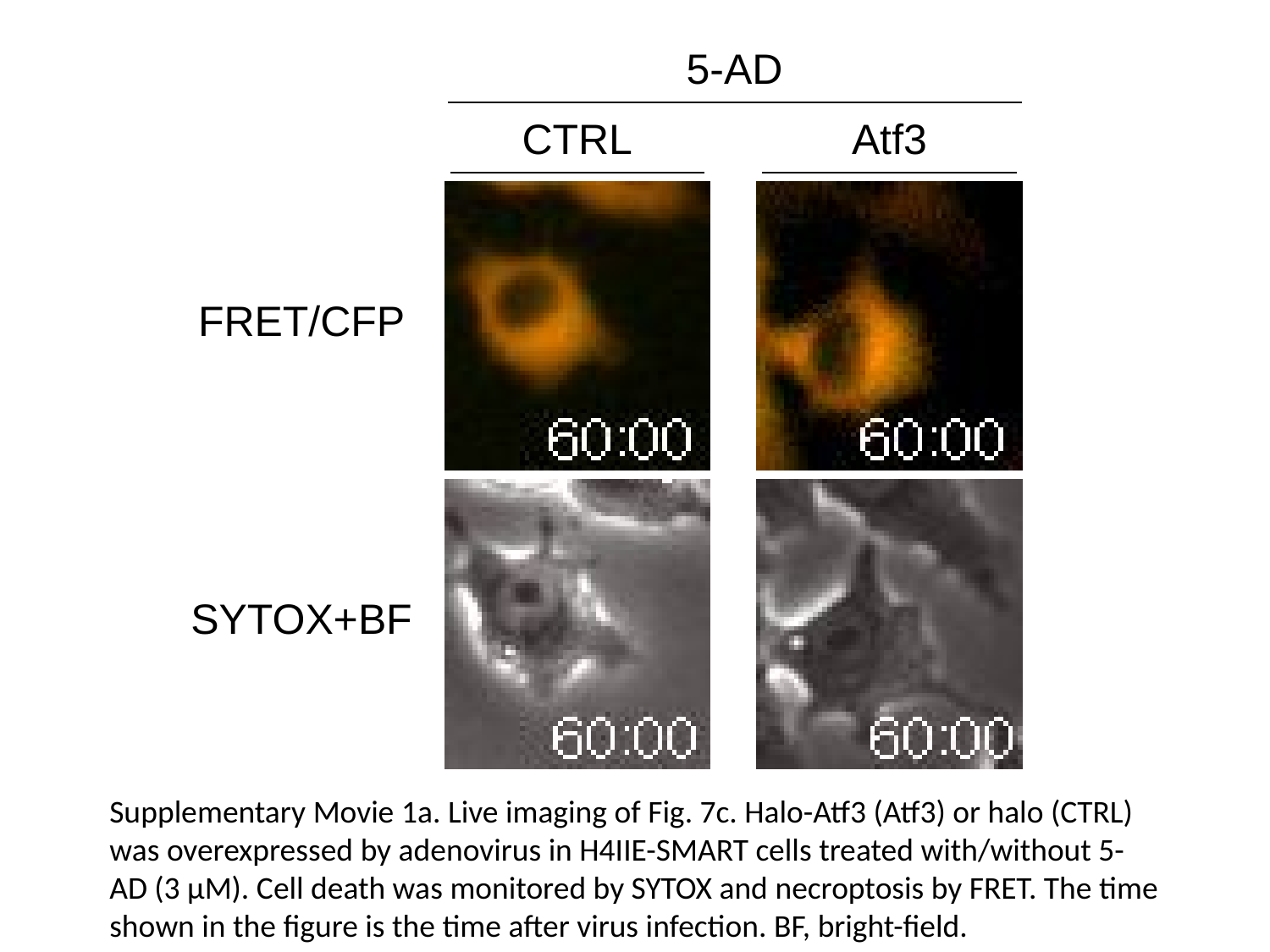

CTRL
Atf3
5-AD
FRET/CFP
SYTOX+BF
Supplementary Movie 1a. Live imaging of Fig. 7c. Halo-Atf3 (Atf3) or halo (CTRL) was overexpressed by adenovirus in H4IIE-SMART cells treated with/without 5-AD (3 μM). Cell death was monitored by SYTOX and necroptosis by FRET. The time shown in the figure is the time after virus infection. BF, bright-field.

## Slide 2
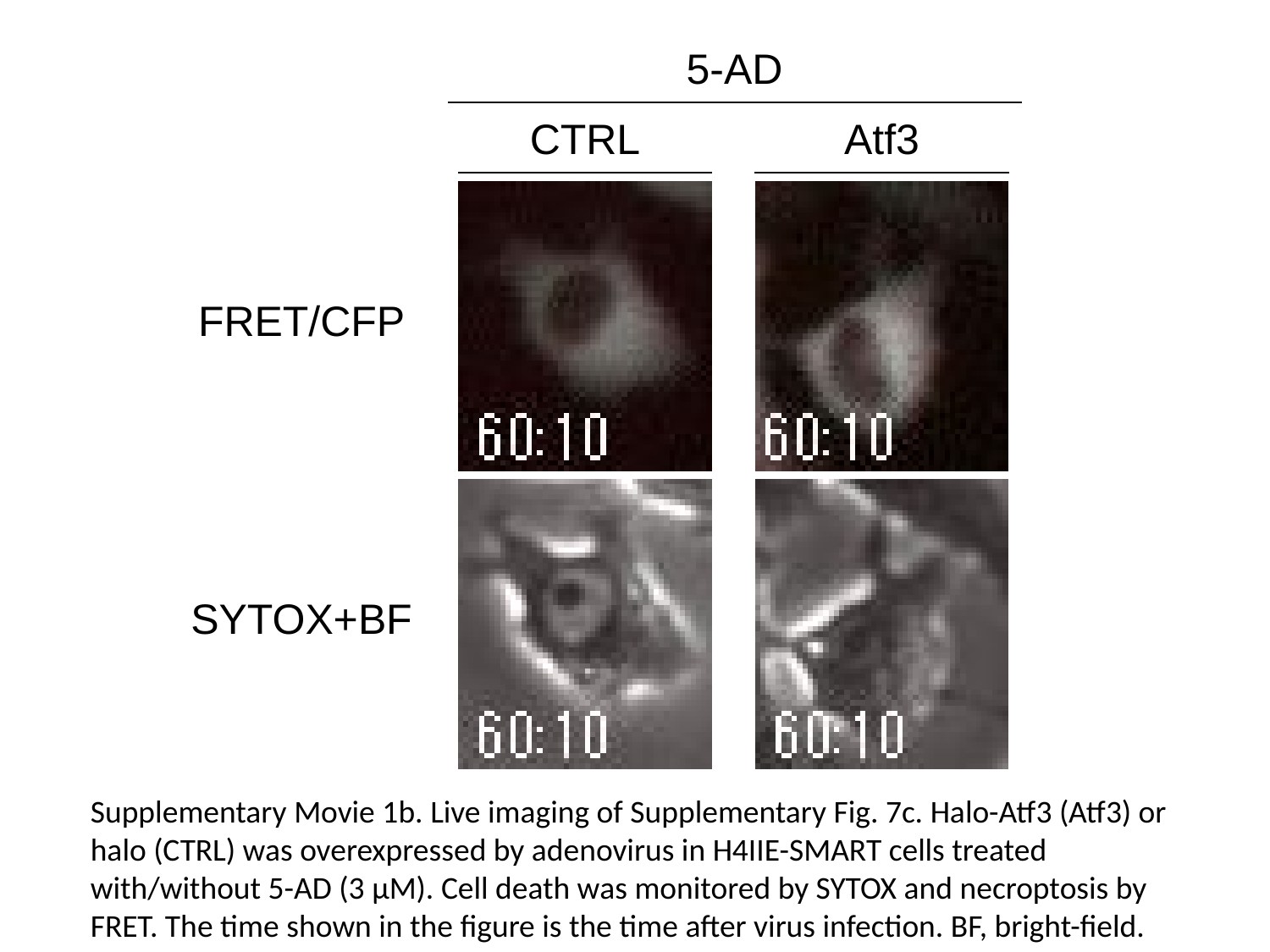

CTRL
Atf3
5-AD
FRET/CFP
SYTOX+BF
Supplementary Movie 1b. Live imaging of Supplementary Fig. 7c. Halo-Atf3 (Atf3) or halo (CTRL) was overexpressed by adenovirus in H4IIE-SMART cells treated with/without 5-AD (3 μM). Cell death was monitored by SYTOX and necroptosis by FRET. The time shown in the figure is the time after virus infection. BF, bright-field.
