# Supplementary material for: The transcription factor ATF3 switches cell death from apoptosis to necroptosis in hepatic steatosis in male mice
Source: Nat Commun. 2023 Jan 23;14:167. doi: 10.1038/s41467-023-35804-w (PMC9871012; doi:10.1038/s41467-023-35804-w)
Supplement: Supplementary file 5 — Supplementary Movie 2 [file 41467_2023_35804_MOESM5_ESM.pptx]

## Slide 1
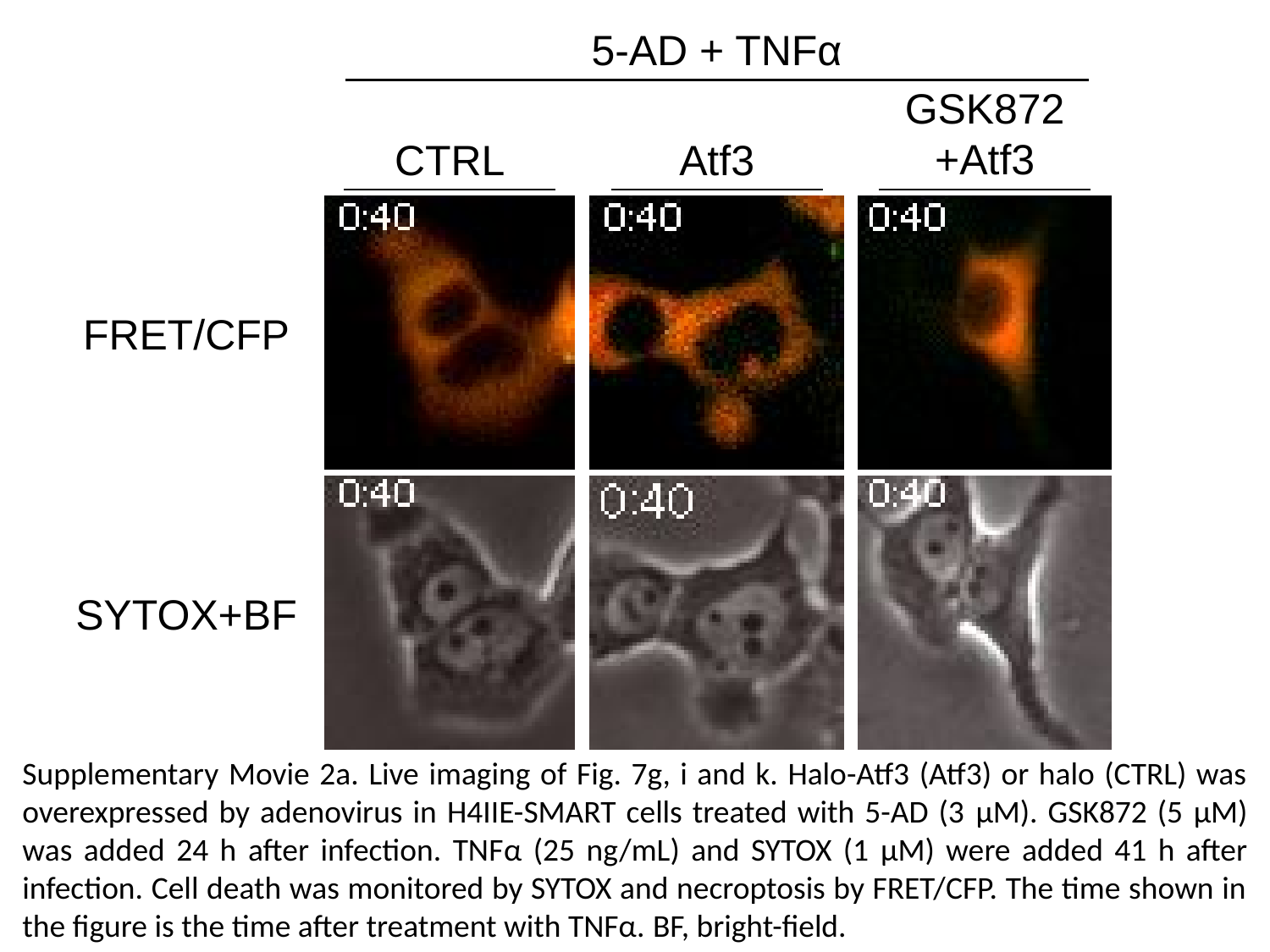

5-AD + TNFα
GSK872
+Atf3
CTRL
Atf3
FRET/CFP
SYTOX+BF
Supplementary Movie 2a. Live imaging of Fig. 7g, i and k. Halo-Atf3 (Atf3) or halo (CTRL) was overexpressed by adenovirus in H4IIE-SMART cells treated with 5-AD (3 μM). GSK872 (5 μM) was added 24 h after infection. TNFα (25 ng/mL) and SYTOX (1 μM) were added 41 h after infection. Cell death was monitored by SYTOX and necroptosis by FRET/CFP. The time shown in the figure is the time after treatment with TNFα. BF, bright-field.

## Slide 2
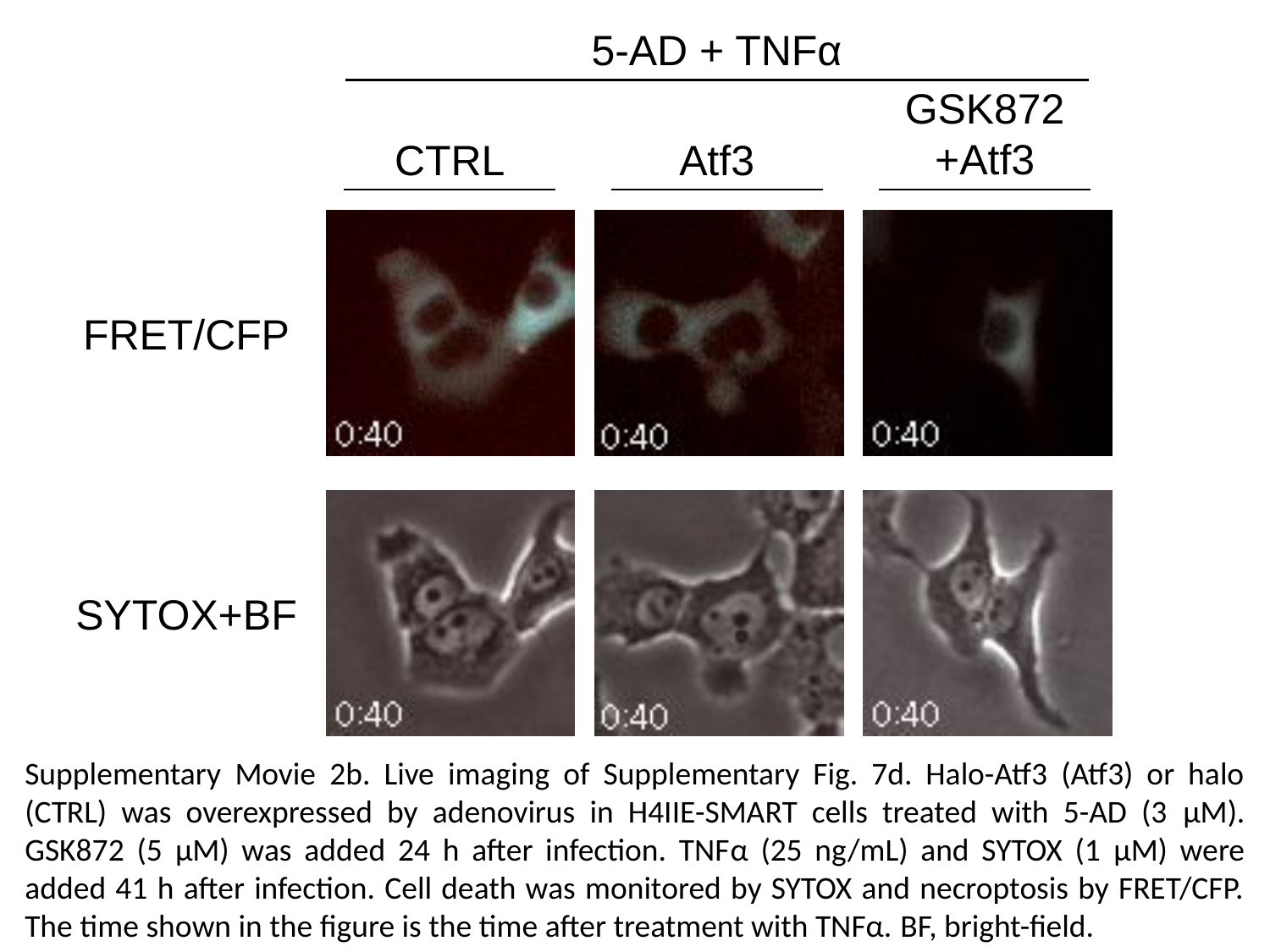

5-AD + TNFα
GSK872
+Atf3
CTRL
Atf3
FRET/CFP
SYTOX+BF
Supplementary Movie 2b. Live imaging of Supplementary Fig. 7d. Halo-Atf3 (Atf3) or halo (CTRL) was overexpressed by adenovirus in H4IIE-SMART cells treated with 5-AD (3 μM). GSK872 (5 μM) was added 24 h after infection. TNFα (25 ng/mL) and SYTOX (1 μM) were added 41 h after infection. Cell death was monitored by SYTOX and necroptosis by FRET/CFP. The time shown in the figure is the time after treatment with TNFα. BF, bright-field.
