# Supplementary material for: The transcription factor ATF3 switches cell death from apoptosis to necroptosis in hepatic steatosis in male mice
Source: Nat Commun. 2023 Jan 23;14:167. doi: 10.1038/s41467-023-35804-w (PMC9871012; doi:10.1038/s41467-023-35804-w)
Supplement: Supplementary file 6 — Reporting Summary [file 41467_2023_35804_MOESM6_ESM.pdf]

nature portfolio

Corresponding author(s): Hiroshi Inoue

Last updated by author(s): Dec 13, 2022

Reporting Summary

Nature Portfolio wishes to improve the reproducibility of the work that we publish. This form provides structure for consistency and transparency in reporting. For further information on Nature Portfolio policies, see our Editorial Policies and the Editorial Policy Checklist.

Statistics

For all statistical analyses, confirm that the following items are present in the figure legend, table legend, main text, or Methods section.

n/a	Confirmed	
☐	☒	The exact sample size (n) for each experimental group/condition, given as a discrete number and unit of measurement
☐	☒	A statement on whether measurements were taken from distinct samples or whether the same sample was measured repeatedly
☐	☒	The statistical test(s) used AND whether they are one- or two-sided<br>*Only common tests should be described solely by name; describe more complex techniques in the Methods section.*
☐	☒	A description of all covariates tested
☐	☒	A description of any assumptions or corrections, such as tests of normality and adjustment for multiple comparisons
☐	☒	A full description of the statistical parameters including central tendency (e.g. means) or other basic estimates (e.g. regression coefficient) AND variation (e.g. standard deviation) or associated estimates of uncertainty (e.g. confidence intervals)
☐	☒	For null hypothesis testing, the test statistic (e.g. F, t, r) with confidence intervals, effect sizes, degrees of freedom and P value noted<br>*Give P values as exact values whenever suitable.*
☒	☐	For Bayesian analysis, information on the choice of priors and Markov chain Monte Carlo settings
☒	☐	For hierarchical and complex designs, identification of the appropriate level for tests and full reporting of outcomes
☐	☒	Estimates of effect sizes (e.g. Cohen's d, Pearson's r), indicating how they were calculated

Our web collection on statistics for biologists contains articles on many of the points above.

Software and code

Policy information about availability of computer code

Data collection	BZ-X700 & Timelups module BZ-H3XT (Keyence, Osaka, Japan)<br>ChemiDoc Touch Imaging System with Bio-Rad CFX Manager V1.5 (Bio-Rad, Hercules, CA)<br>PyroMark Q24 Pyrosequencer in conjunction with PyroMark Q24 Assay software 2.0.6 (Qiagen, Venlo, Netherlands).
Data analysis	Microsoft Excel 2016, IBM SPSS Statistics 24 (IBM Japan, Tokyo, Japan)

For manuscripts utilizing custom algorithms or software that are central to the research but not yet described in published literature, software must be made available to editors and reviewers. We strongly encourage code deposition in a community repository (e.g. GitHub). See the Nature Portfolio guidelines for submitting code & software for further information.

Data

Policy information about availability of data

All manuscripts must include a data availability statement. This statement should provide the following information, where applicable:

- Accession codes, unique identifiers, or web links for publicly available datasets
- A description of any restrictions on data availability
- For clinical datasets or third party data, please ensure that the statement adheres to our policy

Data Availability: The excel sheet with source data are provided with this paper.

Human research participants

Policy information about studies involving human research participants and Sex and Gender in Research.

Reporting on sex and gender	We used liver tissue samples from 43 patients (21 male and 22 female).
Population characteristics	Liver tissue samples were obtained from 48 patients diagnosed with NAFLD through ultrasound-guided percutaneous liver biopsy. All tissue specimens were collected from the hepatobiliary file of the Department of Human Pathology, Graduate School of Medical Sciences, Kanazawa University. Baseline characteristics are presented in Supplementary Table 5.
Recruitment	Patients diagnosed with NAFLD by ultrasound-guided percutaneous liver biopsy were included in the study. Liver biopsy were performed for diagnosing NAFLD/NASH in general medical practice. All patients gave written informed consent to participate in the study in accordance with the Declaration of Helsinki. The remaining liver biopsy samples after NAFLD/NASH diagnosis were used in this study.
Ethics oversight	This study was approved by our institution's ethics committee: Medical Ethics Committee of Kanazawa University, approval no. 2016-072(305).

Note that full information on the approval of the study protocol must also be provided in the manuscript.

Field-specific reporting

Please select the one below that is the best fit for your research. If you are not sure, read the appropriate sections before making your selection.

☒ Life sciences ☐ Behavioural & social sciences ☐ Ecological, evolutionary & environmental sciences

For a reference copy of the document with all sections, see nature.com/documents/nr-reporting-summary-flat.pdf

Life sciences study design

All studies must disclose on these points even when the disclosure is negative.

Sample size	For in vivo and in vitro animal experiments, the sample size for each experiment was calculated based on similar previous studies.<br>The sample size for human data was limited by availability. Published papers was used as references (PMID: 35115492; 31610178; 31517956; 25420998)
Data exclusions	No data were excluded from the analyses.
Replication	Animal experiments were performed with sufficient sample sizes as stated above. Every experiment was repeated at least twice and all attempts to replicate experiments were successful. In vitro experiments were performed in triplicate, repeated at least three times and all attempts to replicate experiments were successful.
Randomization	Before the experiments, mice were grouped according to age and similar body weight. Well location for cell culture experiments were randomly arranged.
Blinding	In some of the animal experiments, the investigators were not blinded because we needed to know whether animals were injected with each siRNA or adenovirus. After collecting the samples, the investigators were blinded to group allocation for data collection and analysis.

Reporting for specific materials, systems and methods

We require information from authors about some types of materials, experimental systems and methods used in many studies. Here, indicate whether each material, system or method listed is relevant to your study. If you are not sure if a list item applies to your research, read the appropriate section before selecting a response.

Materials & experimental systems

n/a	Involved in the study	
☐	☒	Antibodies
☐	☒	Eukaryotic cell lines
☒	☐	Palaeontology and archaeology
☐	☒	Animals and other organisms
☒	☐	Clinical data
☒	☐	Dual use research of concern

Methods

n/a	Involved in the study	
☒	☐	ChIP-seq
☒	☐	Flow cytometry
☒	☐	MRI-based neuroimaging

Antibodies

Antibodies used

The following antibodies were used for the experiments:
Cleaved caspase-3: Cell Signaling #9661S
Caspase-3: Cell Signaling #9665
Cleaved caspase-8: Cell Signaling #8592S
Caspase-8: ENZO ALX-804-447
p-RIPK3 (S232): Abcam ab195117
RIPK3: Abcam ab58828
ACTIN: Sigma-Aldrich A5441
p-eIF2α (S51): Cell Signaling #3597S
eIF2α: Cell Signaling #9722
CHOP: Santa Cruz sc-575
ATF3: Santa Cruz sc-188
p-MLKL (S345): Abcam ab196436
MLKL: Cell Signaling #28640
Halo: Promega G9281
GFP: MBL 598
mCherry: Proteintech 26765-1-AP
mCherry: SICGEN AB0040-200
Cleaved caspase-8: NOVUS NB100-56116
Caspase-8: Proteintech 13423-1-AP
p-RIPK3 (S316): Signalway Antibody #12840
RIPK3: NOVUS NBP1-77299
p-MLKL (S358): Invitrogen PA5-105678
MLKL : Proteintech 66675-1-Ig
GPX4: Abcam ab125006
GADD34: Santa Cruz sc-825
p-c-Jun (S63): Cell Signaling #9261
c-Jun: Santa Cruz sc-1694

The following antibodies were used for human research:
ATF3: Abcam Ab191513
RIPK3: Cell Signaling #10188
pRIPK3 (S227): Abcam Ab209384

Validation

All antibodies were validated by the suppliers.

Cleaved caspase-3 (Asp175): Cell Signaling #9661S, Reactivity: Human, Mouse, Rat, Monkey, Application: WB, IP, IHC, IF, F
Caspase-3 (8G10): Cell Signaling #9665, Reactivity: Human, Mouse, Rat, Monkey, Application: WB, IP
Cleaved caspase-8 (Asp387)(D5B2)XP: Cell Signaling #8592S, Reactivity: Mouse, Application: WB, IP, IF, F
Caspase-8 (1G12): ENZO ALX-804-447, Reactivity: Mouse, Application: ELISA, Flow Cytometry, ICC, WB
p-RIPK3 (S232)[EPR9516(N)-25]: Abcam ab195117, Reactivity: Mouse, Application: ELISA, WB, Dot blot
RIPK3: Abcam ab58828, Reactivity: Mouse, Rat, Application: WB
ACTIN (AC-15): Sigma-Aldrich A5441, Reactivity: sheep, carp, feline, chicken, rat, mouse, Hirudo medicinalis, rabbit, canine, pig, human, bovine, guinea pig, Application: ELISA (i), IF, IHC-P, WB
p-eIF2α (S51)(119A11): Cell Signaling #3597S, Reactivity: Human, Mouse, Rat, Monkey, D. melanogaster, Application: WB, IP, IHC-P
eIF2α: Cell Signaling #9722, Reactivity: Human, Mouse, Rat, Monkey, Application: WB
CHOP (F-168): Santa Cruz sc-575, Reactivity: Mouse, Rat, Human, Application: WB, IF, IHC, ELISA
ATF3 (C-19): Santa Cruz sc-188, Reactivity: Mouse, Rat, Human, Application: WB, IP, IF
p-MLKL (S345): Abcam ab196436, Reactivity: Mouse, Application: WB, IP, Dot Blot
MLKL: Cell Signaling #28640, Reactivity: Mouse, Application: WB
Halo: Promega G9281, Application: WB, IP
GFP: MBL 598, Application: WB, IP, ICC, IHC
mCherry: Proteintech 26765-1-AP, Application: IF, WB, ELISA
mCherry: SICGEN AB0040-200, Application: WB, IF, IHC-P, IHC-Fr
Cleaved caspase-8: NOVUS NB100-56116, Reactivity: Human, Mouse, Rat, Mammal, Application: WB, Flow, ICC/IF, IHC, IHC-Fr, IHC-P, IP
Caspase-8: Proteintech 13423-1-AP, Reactivity: Human, Mouse, Chicken, Pig, Rabbit, Rat, Application: IF, IHC, IP, WB, ELISA
p-RIPK3 (S316): Signalway Antibody #12840, Reactivity: Human, Mouse, Rat, Application: WB
RIPK3: NOVUS NBP1-77299, Reactivity: Human, Mouse, Rat, Application: WB, ELISA, ICC/IF, IHC, IHC-P, IP, Gel Supershift assay
p-MLKL (S358): Invitrogen PA5-105678, Reactivity: Human, Mouse, Rat, Application: IHC-P, WB
MLKL : Proteintech 66675-1-Ig, Reactivity: Human, Mouse, Rat, Application: IF, IHC, WB,ELISA
GPX4: Abcam ab125006, Reactivity: Human, Mouse, Rat, Application: Flow Cyt (Intra), WB, IHC-P, ICC
GADD34 (C-19): Santa Cruz sc-825, Reactivity: Mouse, Rat, Application: WB, IP, IF, Solid phase ELISA
p-c-Jun (S63): Cell Signaling #9261, Reactivity: Human, Mouse, Rat, Monkey, Application: WB, IP, IF, Flow Cytometry
c-Jun (H-79): Santa Cruz sc-1694, Reactivity: Human, Mouse, Rat, Xenopus laevis, Application: WB, IP, IF, IHC-P, Flow Cytometry, Solid phase ELISA

The following antibodies were used for human research:
ATF3: Abcam Ab191513, Reactivity: Human, Application: IHC-P, ICC/IF
RIPK3 (E7A7F): Cell Signaling #10188, Reactivity: Human, Application: WB, IP, IHC-P, IF-IC, FC-FP
pRIPK3 (S227) (EPR9627): Abcam Ab209384, Reactivity: Human, Application: ELISA, Dot blot, WB

Eukaryotic cell lines

Policy information about cell lines and Sex and Gender in Research

Cell line source(s)	H4IIE cells were obtained from ATCC. 293AAV cells were obtained from Agilent.
Authentication	Each cell line was maintained separately and stocked in early passages, to minimize contamination and to preserve cell identity. No further authentication was performed before use.
Mycoplasma contamination	The cell lines tested negative for mycoplasma contamination.
Commonly misidentified lines (See ICLAC register)	No commonly misidentified cell lines were used in this study.

Animals and other research organisms

Policy information about studies involving animals; ARRIVE guidelines recommended for reporting animal research, and Sex and Gender in Research

Laboratory animals	Seven-week-old C57BL/6J mice and C57BL/6JHamSlc-ob/ob mice were obtained from Japan SLC (Shizuoka, Japan). Atf3flox/flox mice were generated previously* and crossed with albumin-Cre mice expressing Cre recombinase in the hepatocytes in order to generate liver-specific Atf3 knockout mice. The littermates were used as controls. Experiments were performed on male mice between the ages of 7 and 25 weeks.<br><br>*Taketani, K., et al. Key role of ATF3 in p53-dependent DR5 induction upon DNA damage of human colon cancer cells. Oncogene 31, 2210-2221 (2012).
Wild animals	No wild animals were used in this study.
Reporting on sex	We used male mice because the prevalence of nonalcoholic fatty liver disease is higher in men than in women.
Field-collected samples	This study did not involve field-collected samples.
Ethics oversight	Mouse experiments were conducted in accordance with the Guide for the Care and Use of Laboratory Animals, eighth edition, and were approved by the Committee for Ethical Use of Experimental Animals of Kanazawa University, Kanazawa, Japan (approval no. AP-194033).

Note that full information on the approval of the study protocol must also be provided in the manuscript.
